# Disorders of fatty acid homeostasis

**DOI:** 10.1002/jimd.12734

**Published:** 2024-05-01

**Authors:** Frédéric M. Vaz, Sacha Ferdinandusse, Gajja S. Salomons, Ronald J. A. Wanders

**Affiliations:** ^1^ Department of Clinical Chemistry and Pediatrics, Laboratory Genetic Metabolic Diseases Emma Children's Hospital, Amsterdam UMC location University of Amsterdam Amsterdam The Netherlands; ^2^ Inborn Errors of Metabolism, Amsterdam Gastroenterology Endocrinology Metabolism Amsterdam The Netherlands; ^3^ Core Facility Metabolomics Amsterdam UMC location University of Amsterdam Amsterdam The Netherlands

**Keywords:** 2‐hydroxylation, fatty acid elongation, mitochondrial disorders, peroxisomal disorders, (phospho)lipid metabolism, sphingolipid metabolism, ω‐hydroxylation

## Abstract

Humans derive fatty acids (FA) from exogenous dietary sources and/or endogenous synthesis from acetyl‐CoA, although some FA are solely derived from exogenous sources (“essential FA”). Once inside cells, FA may undergo a wide variety of different modifications, which include their activation to their corresponding CoA ester, the introduction of double bonds, the 2‐ and ω‐hydroxylation and chain elongation, thereby generating a cellular FA pool which can be used for the synthesis of more complex lipids. The biological properties of complex lipids are very much determined by their molecular composition in terms of the FA incorporated into these lipid species. This immediately explains the existence of a range of genetic diseases in man, often with severe clinical consequences caused by variants in one of the many genes coding for enzymes responsible for these FA modifications. It is the purpose of this review to describe the current state of knowledge about FA homeostasis and the genetic diseases involved. This includes the disorders of FA activation, desaturation, 2‐ and ω‐hydroxylation, and chain elongation, but also the disorders of FA breakdown, including disorders of peroxisomal and mitochondrial α‐ and β‐oxidation.

## 1 INTRODUCTION

Fatty acids (FA) are hydrocarbon chains that terminate in a carboxylic acid group (–COOH) and serve a great number of physiological functions. The different types of FA classes shown in this review are summarized in Table 1. First, FA are an important source of energy. To this end, FA are cleaved into a series of acetyl units by the mitochondrial and peroxisomal β‐oxidation systems, after which the acetyl units are either degraded to CO_2_ and H_2_O in mitochondria via the concerted action of the tricarboxylic (Krebs) cycle and the mitochondrial oxidative phosphorylation system or used for the biosynthesis of the ketone bodies 3‐hydroxybutyrate and acetoacetate in the liver to be oxidized in extrahepatic tissues. Second, FA are important building blocks of membrane lipids and thus contribute to the specific barrier function of biological membranes. The mitochondrial inner membrane, for instance, is a unique membrane that is even impermeable to protons. This property allows the mitochondrion to build up a large proton electrochemical gradient upon the transfer of electrons through the respiratory chain, which is the driving force behind the synthesis of ATP. The permeability barrier imposed by biological membranes also allows compartmentalization of different processes within cells and allows different intracellular organelles to perform distinct functions in cellular metabolism. Third, FA are important signaling molecules either as such or after conversion into some FA‐derived metabolites. Indeed, FA are ligands of several G protein‐coupled receptors, including FFAR1/GPR40 and FFAR4/GPR120.^1^ Furthermore, intracellular FA, notably long‐chain FA (LCFA), are ligands of a group of nuclear hormone receptors called the peroxisome proliferator‐activated receptors (PPAR) of which there are three in humans, including PPARα, β/δ, and γ, each with a different tissue distribution and physiological role.^2^ Apart from the signaling role of free FA (FFA), there are many examples of FA‐derived signaling molecules, which include lysophospholipids like lysophosphatidic acid and *N*‐acyl taurines (NAT) like C22:6‐NAT.^3^ Yet another level of cellular signaling by FA involves the N‐posttranslational modification of proteins by acyl‐CoAs, notably *N*‐myristoylation and *N*‐palmitoylation (see Martin et al.^4^ for review). Finally, FA are precursors of a plethora of different lipid mediators, including the classical eicosanoids, which comprise prostaglandins, thromboxanes, and leukotrienes, but also more recently identified molecules like the resolvins, protectins, and maresins, which all activate their own specific receptors and by doing so, exert multiple physiological functions.^5^

**TABLE 1:** Overview of different fatty acid classes.

Example FA and chemical structure		Abbreviation		FA Class
Butyric acid	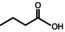	C4:0	SCFA	Short‐chain FA C2–C4
Octanoic acid	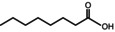	C8:0	MCFA	Medium‐chain FA C5–C10
Palmitic acid	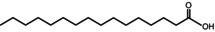	C16:0	LCFA	Long‐chain FA C11–C20
Oleic acid	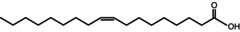	C18:1ω6	MUFA	Monounsaturated FA
Arachidonic acid	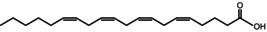	C20:4ω6	PUFA	Polyunsaturated FA
Cerebronic acid	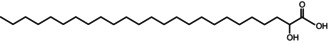	2‐OH‐C24:0	2‐OH‐FA	2‐Hydroxy FA
ω‐OH‐lignoceric acid	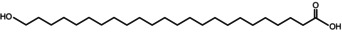	ω‐OH‐C24:0	ωOH‐FA	ω‐Hydroxy FA
Cerotic acid	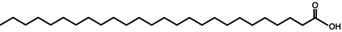	C26:0	VLCFA	Very long‐chain FA >C20
Melissic acid	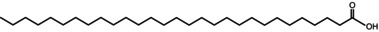	C30:0	ULCFA	Ultra long‐chain FA >C26
Phytanic acid 3,7,11,15‐tetramethylhexadecanoic acid	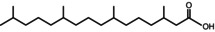	C20:0	3MeBCFA	3‐Methyl branched‐chain FA
Pristanic acid 2,6,10,14‐tetramethylpentadecanoic acid	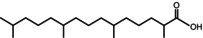	C19:0	2MeBCFA	2‐Methyl branched‐chain FA

**FIGURE 1:**
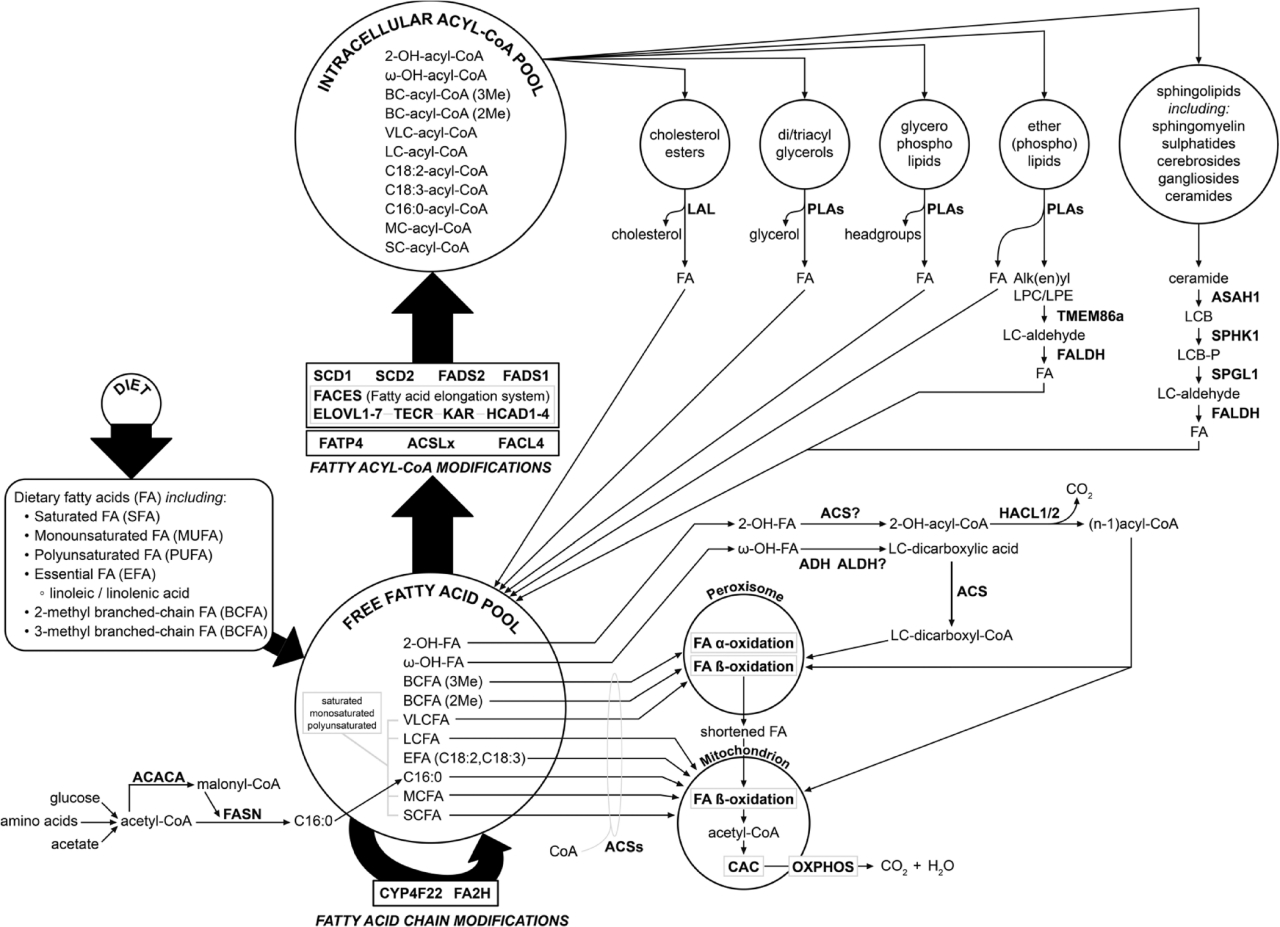
Overview of the essential features of fatty acid (FA) homeostasis in humans. FA may be derived from exogenous, dietary sources, and/or synthesized endogenously from acetyl‐CoA, except for the essential FA which can only be obtained from exogenous sources. The free FA pool thus obtained can then undergo a range of modifications ultimately generating an extended acyl‐CoA ester pool which can then be used for the synthesis of multiple lipid species which serve different roles in human physiology. As part of each lipid species' life cycle, FA are released once again from the different lipid species to reenter the free FA pool where they can be either reutilized or undergo full oxidation to CO_2_ and H_2_O via FA oxidation in peroxisomes and/or mitochondria. See text for more information.

In humans, FA are derived from exogenous dietary sources but can also be synthesized endogenously, although the FA repertoire synthesized endogenously from acetyl‐CoA units by means of the cytoplasmic enzyme complex fatty acid synthase (FASN) is much more limited compared to the spectrum of dietary FA. This is due to the fact that humans lack the capacity to introduce double bonds beyond the Δ^9^ position in FA chains, which explains why linoleic acid (*cis*, *cis*‐Δ^9^,Δ^12^‐C18:2) and linolenic acid (all *cis*‐Δ^9^,Δ^12^,Δ^15^‐C18:3) are essential FA required for the synthesis of polyunsaturated FA (PUFA). The end product of the FASN complex is palmitic acid (Figure 1). After activation by coenzyme A, the resulting palmitoyl‐CoA can undergo chain elongation via the FA chain elongation system (FACES) either directly or after prior Δ^9^‐desaturation to produce a series of monounsaturated FA (MUFA), as discussed later in this review.

Dietary FA present in our daily food are either esterified or present as FFA. Through the concerted action of different lipases, including pancreatic and intestinal lipases, all esterified FA are released as FFA and subsequently re‐esterified in the intestinal mucosa and incorporated into chylomicrons. The lymphatic system then delivers these lipoproteins to the general circulation, followed by the lipoprotein lipase‐mediated release of FA and subsequent delivery and metabolism of the various FA in different tissues. Medium‐chain FA (MCFA) are not incorporated into chylomicrons and are directly targeted to the liver via the portal vein.

In plasma, the total FFA concentration ranges from 100 to 400 μmol/L. The high affinity of albumin for FFA, together with its marked abundance amounting to 300–600 μmol/L, assures that the concentration of non‐protein bound FFA is very low, amounting to some 1–10 nmol/L.^6^ Once inside the cell, FFA are bound by one of the nine different fatty acid binding proteins (FABP), each showing a different tissue distribution.^7^ Indeed, for hepatocytes and cardiomyocytes, the free, non‐protein‐bound concentration of FFA has been estimated to be 1–5 nmol/L, whereas the total FFA concentration may be up to 50 μmol/L. Albumin and the different FABPs thus constitute a powerful buffer system for FFA thereby limiting the cytotoxic effects of FFA.

The cellular uptake of FFA has long been disputed. Indeed, although it was long believed that FFA would enter cells freely, there is now consensus that FFA uptake is, in fact, protein‐mediated, with CD36 (SR‐B2) as the main gatekeeper of FFA transport. Current knowledge holds that the presence of CD36 at the plasma membrane is subject to strict regulation by virtue of the fact that, at least in cardiomyocytes, CD36 reversibly recycles between endosomes (intracellular storage) and the sarcolemma thereby regulating the rate of FFA entry much in the same way as glucose transport is controlled by GLUT4.^8^

FA occur in a bewildering number of different forms and shapes (see Table 1), which is caused on the one hand by the fact that the FA that humans obtain from dietary sources themselves already are heterogeneous in structure, but in addition that all FFA either de novo synthesized or derived from dietary sources can also undergo a range of modifications, further expanding the FA repertoire. These modifications include activation to a CoA ester, the introduction of double bonds at certain positions, as well as the 2‐hydroxylation and ω‐hydroxylation of FA, and the elongation of FA via the FACES (see Figure 1). The importance of these FA modifications is stressed by the existence of a range of genetic diseases in man caused by variants in genes coding for such modifying enzymes, as discussed below. Most of these modification reactions involve acyl‐CoA esters rather than FFA species. In contrast, the activation, 2‐hydroxylation, and ω‐hydroxylation of FA involve FFA as substrates, which implies that in the case of 2‐hydroxylation and ω‐hydroxylation of FFA, activation to the corresponding acyl‐CoA esters occurs *after* the modification reaction (see Figure 1). Taken together, the result of all these modifications inserted into the different FA species taken up from our daily diet as well as synthesized endogenously is an extended acyl‐CoA ester pool that can be used for the synthesis of many different lipid species, including cholesterol esters, di‐ and triacylglycerols, glycerophospholipids, sphingolipids (SL), and ether(phospho)lipids (see Figure 1).

Homeostasis dictates that there is not only biosynthesis of all the different FA species to be incorporated into the various lipid species but also degradation. Indeed, each of the main classes of FA‐containing lipid species, as shown in Figure 1, undergo degradation either in the lysosome or otherwise (to be discussed later), and the FA released during this process join with the FA derived from the diet and synthesized endogenously to form the intracellular FA pool. To keep the FFA pool in check, FA need to be degraded, which occurs via two different mechanisms, including α‐ and β‐oxidation. Saturated as well as MUFA and PUFA can be oxidized by β‐oxidation, both in mitochondria and in peroxisomes, although the two organelles exhibit different substrate specificities. Oxidation of 3‐methyl‐branched‐chain FA, like phytanic acid, is fully dependent on peroxisomes since the enzymatic machinery involved in α‐oxidation is strictly peroxisomal.

In this review, we describe the current state of knowledge about FA homeostasis and the inherited metabolic diseases involved. This includes the disorders of FA biosynthesis, such as the disorders of FA desaturation, 2‐ and ω‐hydroxylation and chain elongation, but also the disorders of FA degradation, which includes the disorders of peroxisomal α‐ and β‐oxidation and mitochondrial β‐oxidation. First, we describe the different FA‐modifying enzyme systems.

## 2 FA HOMEOSTASIS AND THE IMPORTANT ROLE OF FA MODIFICATIONS

The FA derived from dietary sources, synthesized de novo, or generated endogenously by the breakdown of various lipids may undergo a range of different modifications before being incorporated into complex lipids. These modifications include distinct FA hydroxylations, notably for the synthesis of SL, including sphingomyelin, ceramides, gangliosides, and sulfatides, as well as chain elongations and desaturations to generate the multitude of LCFA, very long‐chain LCFA (VLCFA), and ultra LCFA (ULCFA), both saturated as well as monounsaturated and polyunsaturated, especially for incorporation into glycero(phospho)lipids. The essentials of the FA modifications in humans are described below:

### 2.1 FA 2‐hydroxylation

**FIGURE 2:**
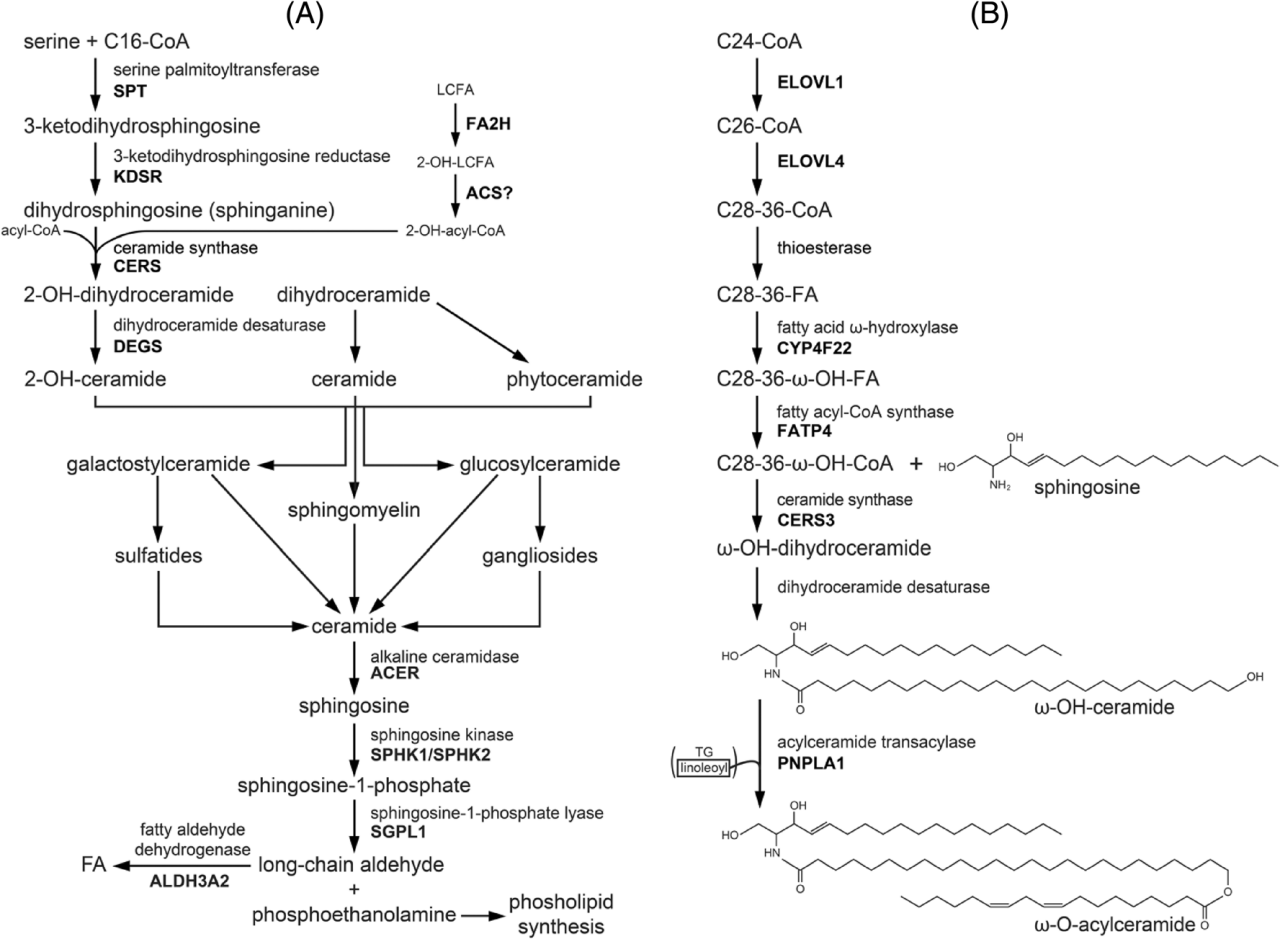
Overview of synthesis and breakdown of different sphingolipids. (A) Biosynthesis of sphingolipids starts with the condensation of serine and palmitoyl‐CoA to generate 3‐keto‐dihydrosphingosine as catalyzed by the enzyme serine palmitoyltransferase which is then converted into ceramide, the unique building block for the synthesis of glucosylceramide and gangliosides, galactosylceramide and sulfatides, and sphingomyelin. These different sphingolipid species are degraded predominantly in lysosomes thereby regenerating ceramide. Ceramide is subsequently broken down by the alkaline ceramidase ACER3 followed by phosphorylation of sphingosine and the other LCB to produce the corresponding LCB‐1‐phosphates which are then cleaved into phosphoethanolamine plus the different long‐chain aldehydes. Finally, ALDH3A2, the enzyme deficient in Sjögren–Larsson syndrome (SLS) will convert the different aldehydes into the corresponding FA for reutilization as is phosphoethanolamine for phospholipid synthesis. (B) Synthesis of ω‐*O*‐acylceramides: after elongation of VLCFA to ULCFA, CoA is removed and CYP4F22 hydroxylates the FFA at the ω‐position, followed by reactivation to the corresponding ω‐OH‐acyl‐CoA ester by FATP4. This CoA ester then condenses with sphingosine, as catalyzed by CERS3, to form ω‐OH‐dihydroceramide. The sphingoid base of the dihydroceramide can be modified yielding ω‐OH‐forms of 6‐OH‐dihydroceramide or phytoceramide. Desaturation of ω‐OH‐dihydroceramide forms ω‐OH‐ceramide, followed by the PNPLA1‐catalyzed transfer of a linoleyl‐side chain from triacylglycerol to the ω‐OH group of ω‐OH group to for ω‐*O*‐acylceramides.

2‐Hydroxy FA (2‐OH‐FA) are found in all kingdoms of eukaryotes and in eubacteria. In higher eukaryotes, 2‐OH‐FA are major constituents of ceramides and SL derived from them, and these 2‐OH‐FA‐SL are especially abundant in some tissues, including the brain, notably in myelin, and skin (see Hama^9^ for review). Indeed, although the FA moiety of ceramides is not hydroxylated in most tissues, ceramides with 2‐OH‐FA do exist in specific tissues such as the epidermis and brain. At present, the only known enzyme that hydroxylates FA at the 2‐position is the enzyme FA 2‐hydroxylase (FA2H), which synthesizes the (2R)‐enantiomer. Interestingly, 2‐OH‐FA also occur in bacteria but only as (2S)‐enantiomers, and these (2S)‐enantiomers are present in certain food components. The enzyme FA2H is an NADPH‐dependent monooxygenase localized in the endoplasmic reticulum (ER) membrane with its catalytic site facing the cytosol and belongs to the family of FA hydroxylases/desaturases. Figure 2A depicts the biosynthesis of ceramides and sphingomyelins and the key role of FA2H in generating the 2‐OH‐FA, which, after activation to the corresponding 2‐OH‐acyl‐CoA, enter the biosynthetic route of 2‐OH‐FA‐containing ceramides and sphingomyelins (see Eckhardt^10^ for a recent review).

Although FFA rather than acyl‐CoA esters have been shown to be substrates of FA2H,^11^ the true substrate specificity of FA2H has remained ill‐defined. Importantly, available evidence indicates that FA2H is surely not the only enzyme capable of synthesizing 2‐OH‐FA. This is concluded from work in mice lacking a functional *Fa2h* gene. These mice are completely devoid of 2‐OH‐FA‐containing SL in the nervous system but not in various other organs, including the skin.^12^ There is at least one other enzyme in mammals able to 2‐hydroxylate FA. This is the enzyme phytanoyl‐CoA 2‐hydroxylase (PHYH), which only accepts acyl‐CoA esters (but not FFA) and is strictly localized in peroxisomes (see Wanders et al.^13^ for review). Although it remains to be established whether PHYH is generating the 2‐OH‐FA in other tissues, including the skin, work by Mukherji et al.^14^ has shown that PHYH is indeed reactive with a range of saturated unbranched acyl‐CoAs, including palmitoyl‐CoA. 2‐OH‐FA synthesized by FA2H are subsequently activated to the corresponding CoA ester by one or more of the 26 acyl‐CoA synthetases expressed in humans^15^ and used for the formation of 2‐OH‐FA‐SL. Ceramide synthases, of which there are six in humans^16^ (CERS1‐6), accept acyl‐CoAs as well as 2‐OH‐acyl‐CoAs as substrate, as shown for all ceramide synthases by Mizutani et al.^17^

The functional role of 2‐OH‐FA‐SL in biological membranes has remained enigmatic, although they do appear to play a role in membrane trafficking and stabilization of membrane microdomains. In the mammalian nervous system, the majority of 2‐OH‐FA‐SL are 2‐OH‐FA‐galactosylceramides and 2‐OH‐FA‐sulfatides, which are abundant constituents of myelin. Studies in *Fa2h*‐deficient mice have shown that 2‐OH‐FA‐SL are not so much essential for building the myelin sheaths themselves but are required for long‐term myelin maintenance and axonal support.^18^, ^19^ Skin also contains large amounts of 2‐hydroxylated ceramides and glucosylceramides, but the specific role of these 2‐hydroxylated lipids is unclear.^10^

#### 2.1.1 FA2H deficiency

Biallelic variants in *FA2H* have been described in three clinically defined disease entities, including leukodystrophy with spasticity and dystonia, neurodegeneration with brain iron accumulation (NBIA), and hereditary spastic paraplegia (HSP) type SPG35. Recently, Schüle et al. performed a detailed study in a cohort of 19 genetically confirmed cases and observed that the clinical phenotype is, in fact, rather uniform with lower limb‐predominant spastic‐tetraparesis accompanied by truncal instability, dysarthria, dysphagia, cerebellar ataxia, and cognitive defects in ~90% of cases often accompanied by exotropia and movement disorders.^20^ Disease progression is rapid with loss of ambulation (median time after onset of disease: 7 years). A unique feature of FA2H deficiency is the wiry hair having a bristle‐like appearance which compares well with similar findings in a mouse model of FA2H deficiency.^12^ The MRI phenotype was also strikingly uniform, and the findings are described by the so‐called “WHAT” acronym, which includes *W*hite matter changes, *H*ypointensity of the globus pallidus, pontocerebellar *A*trophy, and a *T*hin corpus callosum. The diagnosis of FAHN/SPG35 due to *FA2H* variants is solely based on molecular analysis with thus far no contribution from biochemical genetics. The rapid progression in the field of lipidomics surely holds promise in this respect.

### 2.2 FA ω‐hydroxylation

Another important FA side‐chain modification concerns the ω‐oxidation of FA required for the synthesis of ω‐OH‐acylceramides, which play an essential role in the skin by imposing a powerful permeability barrier that protects the organism against infectious diseases and allergies by blocking the invasion of external substances such as pathogens, allergens, and chemicals. The epidermis‐specific lipid class of ω‐*O*‐acylceramides plays a crucial role in the formation of the permeability barrier, and abrogation of its synthesis causes the skin disorder ichthyosis. The stratum corneum contains a multilayered lipid structure (the lipid lamellae) mainly composed of ceramides, FA, and cholesterol. Ceramides are the backbone of SL and consist of two hydrophobic chains, including a long‐chain base (LCB) and an *N*‐acyl chain, and occur in a specialized form in the epidermis, that is, ω‐*O*‐acylceramides which differ from regular ceramides in two aspects: (1) the *N*‐acyl chain in ω‐OH‐ceramides is ultra‐long (C28–C36) and has an ω‐OH group at its end instead of a methyl group as in regular ceramides and (2) linoleic acid is usually esterified to the ω‐OH group thereby creating a unique structure consisting of three hydrophobic chains. The enzyme PNPLA1 has been identified as the unique transacylase that specifically transfers linoleic acid from triacylglycerols to the ω‐hydroxy position in ceramides thus giving rise to ω‐acylceramides^21^, ^22^ (see Figure 2B). The unique structure of these ω‐*O*‐acylceramides is essential for both the formation and maintenance of lipid lamellae and skin barrier formation, as concluded from the various IEMs caused by pathogenic variants in the genes involved, as well as studies in mutant mice.^23^ Recent work by Opálka et al. using in vitro multilamellar lipid models clearly showed that ω‐*O*‐acylceramides but not ω‐OH‐ceramides are required for the lamellar phase architecture of skin barrier lipids.^24^

The enzymology of the ω‐*O*‐acylceramide synthesis pathway has been nicely worked out by Kihara (see Figure 2B and Kihara^23^). Inspired by earlier findings, which led to the discovery of a new cytochrome P450 gene mutated in patients affected by lamellar ichthyosis (LI) Type 3,^25^ Ohno et al. identified the cytochrome P450 enzyme CYP4F22 as the long‐sought FA hydroxylase required for ω‐*O*‐acylceramide formation.^26^ The enzyme involved is a typical Type I ER membrane protein that spans the membrane only once, with its catalytic site facing the cytosol. Furthermore, the enzyme was found to have a very restrictive substrate preference, only accepting free ULCFAs (C30:0 > C30:1 > C34:1 > C32:0 > C30:1) as substrate but not the corresponding acyl‐CoAs.^26^ Subsequently, the ULCFA synthesized by CYP4F22 needs to be coupled to the NH_2_ group of different LCB. Epidermal ceramides contain four types of LCB, including dihydrosphingosine (sphinganine), sphingosine, phytosphingosine, and 6‐hydroxysphingosine. The formation of the amide bond between an LCB and ULCFA‐CoA is catalyzed by the enzyme ceramide synthase 3 (CERS3). Since CERS3 only accepts acyl‐CoA esters rather than FFA as substrate, the ULCFA synthesized by CYP4F22 needs to be activated to its corresponding CoA ester. Work again by Yamamoto et al. has identified FATP4/ACSVL4 as the enzyme involved,^27^ which fits with the findings in *Fatp4* knockout mice, which exhibit severe skin barrier dysfunction and epidermis aberrations.^23^

#### 2.2.1 CYP4F22 deficiency

CYP4F22 deficiency belongs to the group of autosomal recessive congenital ichthyosis (ARCI), which is a heterogeneous collection of rare disorders of keratinization characterized by generalized abnormal scaling of the skin. The main skin phenotypes of ARCI are LI, congenital ichthyosiform erythroderma, and harlequin ichthyosis. The genetic basis of ARCI is heterogeneous, with >10 different genes identified. CYP4F22 deficiency was first described in 2006 by Lefèvre et al. in a series of 21 patients with erythroderma, severe scaling being more pronounced on flexural areas, and palmoplantar hyperlinearity.^25^ This was at a time when the functional role of CYP4F22 in the formation of ω‐*O*‐acylceramides was not known. The phenotypic spectrum of CYP4F22 deficiency was enlarged by the identification of two patients born as collodion babies who improved over time with little to no skin abnormalities. This minor variant is now known as “self‐improving collodion ichthyosis.”^28^ The group of Hotz et al. studied a cohort of 770 families with a clinical diagnosis of ARCI and identified pathogenic variants in 54 families,^29^ which expanded the known clinical and molecular spectrum of patients with ARCI due to *CYP4F22* variants. Again, the diagnosis of CYP4F22 deficiency is purely based on genetic testing. Biochemical methods, including lipidomics, would be very helpful, especially since the genetic basis of CYP4F22 deficiency is very heterogeneous, and the interpretation of new variants identified in ARCI patients is not always straightforward.

### 2.3 FA activation

The family of FA‐activating enzymes includes some 26 different members which differ with respect to their tissue distribution, subcellular localization, substrate specificities, and regulation.^30^ At present, deficiencies in only two FA‐activating enzymes have been identified, as described below.

#### 2.3.1 FACL4 deficiency

FACL4 deficiency was first described in a family with Alport syndrome, elliptocytosis, and mental retardation as caused by a contiguous deletion encompassing *COL4A5*.^31^ This was soon followed by a report documenting biallelic, apparently pathogenic variants in *FACL4* in patients from two different families with non‐specific mental retardation.^32^ The diagnosis of FACL4 deficiency was supported by enzymatic studies in lymphocytes, which revealed low acyl‐CoA synthetase activity with arachidonic acid (AA) as substrate. Subsequently, a third family with four affected males was reported carrying a new variant in *FACL4*.^33^ Since then, FACL4 deficiency has only been described in patients carrying different contiguous deletions in the Xq22.3‐q23 region.

#### 2.3.2 FATP4/ACSVL4/SLC27A4 deficiency

Ichthyosis prematurity syndrome (IPS) is one of the syndromic forms of ichthyosis and is characterized by premature birth, neonatal asphyxia, and ichthyosis, which improves during the first weeks of life but persists for life, often accompanied by atopic dermatitis and recurrent infections. The causative gene of IPS is *FATP4*, also known as *ACSVL4* or *SLC27A4*, as first reported by Klar et al.,^34^ with several additional patients reported since then.^35^, ^36^, ^37^, ^38^, ^39^, ^40^ Work by Yamamoto et al. has resolved the functional role of FATP4. Indeed, these workers identified FATP4 as the enzyme activating ω‐OH‐FA to the corresponding ω‐OH‐acyl‐CoAs thus playing an essential role in the formation of ω‐*O*‐acylceramides as exemplified by the results obtained in *Fatp4* knockout mice.^27^

### 2.4 FA desaturation and elongation

**FIGURE 3:**
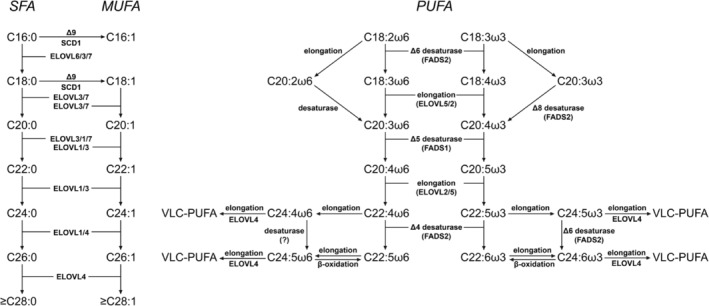
Overview of the synthesis and elongation of both saturated, monounsaturated and polyunsaturated FA to VLCFA and VLC‐PUFA. Elongation of saturated fatty acid (SFA), monounsaturated fatty acid (MUFA), and polyunsaturated fatty acid (PUFA): The process involves initial activation to CoA derivatives, followed by sequential addition of two carbon units from malonyl‐CoA. For SFA, straightforward chain extension occurs. MUFA elongation involves desaturation before or after elongation steps. PUFA undergoes both elongation and additional desaturations, adding double bonds. The resulting elongated fatty acids vary in chain length and degree of unsaturation, contributing to diverse biological functions. See text for more information.

Another major modification of FA concerns their chain elongation, which is often coupled to specific desaturations with the specific purpose to synthesize PUFA (see Figure 3), from which a wide range of bioactive lipids are produced, including eicosanoids, docosanoids, and elovanoids generated from AA (C20:4ω6), eicosapentaenoic acid (C20:5ω3), docosahexaenoic acid (DHA) (C22:6ω3), and very long‐chain PUFA (VLC‐PUFA). Importantly, all enzymes involved with FA desaturation and chain elongation react with acyl‐CoA species and not with FFA. Since mammals, including humans, lack the capacity to introduce double bonds beyond the Δ^9^ position, linoleic acid (C18:2ω6) and α‐linolenic acid (C18:3ω3), which contain double bonds beyond Δ^9^ at positions Δ^12^ and Δ^15^, are essential FA to be derived from dietary sources (see Figure 3).

Desaturation of FA: Humans contain different enzymes able to introduce double bonds in FA, which belong to two distinct families, including the Stearoyl‐CoA desaturase (SCD)^41^ and fatty acid desaturase (FADS) family.^42^ The principle SCD in humans is SCD1, which catalyzes the Δ^9^‐desaturation of the two saturated FA (SFA) palmitate (C16:0) and stearate (C18:0) to the corresponding MUFAs palmitoleate (C16:1ω7) and oleate (C18:1ω9) (see Figure 3A). Human SCD1 deficiency has not been described so far, and much of the conclusions drawn about SCDs have been derived from mouse studies despite the fact that mice express five different SCDs, whereas humans express only two SCDs, that is, SCD1 and SCD5. Much more is known about the different members of the FADS family. Humans express three different FADS enzymes, that is, FADS1, FADS2, and FADS3, and the genes encoding these enzymes are clustered on chromosome 11 with FADS1 and FADS2 arranged head‐to‐head upstream of FADS3. FADS1 and FADS2 were originally identified as catalyzing Δ^5^‐desaturation and Δ^6^‐desaturation, respectively. More recent evidence indicates that FADS2 is the so‐called “general” desaturase equipped with Δ^6^, Δ^4^, and Δ^8^ activity, whereas FADS1 is now considered the “specific” desaturase with Δ^5^‐ and Δ^7^‐desaturase activity and a much more restricted reactivity toward different FA compared to FADS2.^42^ Recent work by Karsai et al. has shed light on the function of FADS3 which was identified as a Δ14Z sphingoid base desaturase, thus indicating that FADS1 and FADS2 are the sole desaturases with activity toward LC‐PUFAs.^43^

**FIGURE 4:**
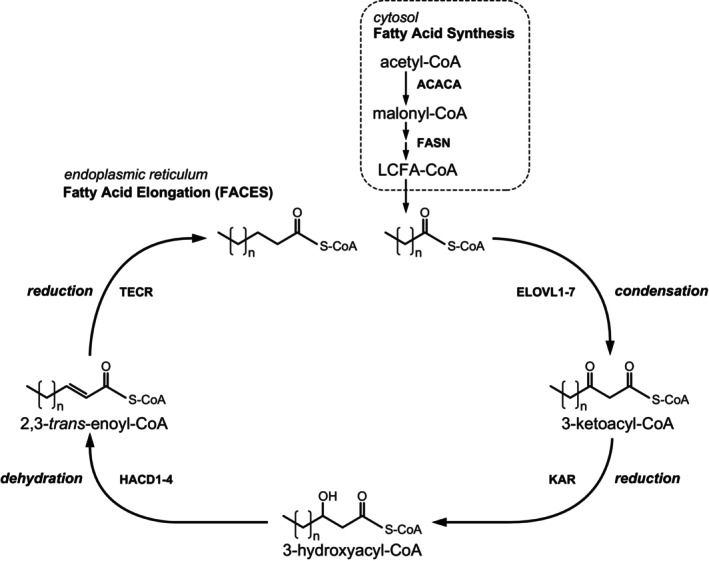
Synthesis and elongation of fatty acids. In the cytoplasm, fatty acids are produced from malonyl‐CoA by the action of the fatty acid synthase complex (FASN), leading to the creation of long‐chain acyl‐CoA (LCFA‐CoA). This LCFA‐CoA is further lengthened in the endoplasmic reticulum (ER), producing an array of very long‐chain fatty acids. The initial phase involves the condensation of malonyl‐CoA with LCFA‐CoA, a process facilitated by a group of enzymes known as elongation of very long‐chain fatty acids (ELOVLs), which exhibit varying patterns of tissue expression and substrate preferences based on the chain length and saturation level of the fatty acid. The 3‐keto group is then converted into a hydroxyl group by 3‐ketoacyl‐CoA‐reductase (KAR). Subsequently, the formed 3‐hydroxyacyl‐CoA undergoes dehydration by a series of 3‐hydroxyacyl‐CoA dehydratases (HACD1‐4), each with distinct patterns of tissue distribution, to produce 2,3‐*trans*‐enoyl‐CoA. In the final step, the double bond in this molecule is hydrogenated by *trans*‐2,3‐enoyl‐CoA reductase (TECR), resulting in an acyl‐CoA molecule that is elongated by two carbon units.

FA chain elongation: The FACES is a four‐step pathway that results in the net addition of two carbon atoms to the FA chain (see Figure 4). In principle, the reaction mechanism is identical to that of the peroxisomal and mitochondrial β‐oxidation systems but then in the opposite direction. As a result, two carbon atoms are added to the FA chain per elongation cycle. The mechanism behind these two opposing machineries has been resolved and is explained by the finding that the first and third steps of β‐oxidation are driven by NAD^+^‐dependent enzymes, whereas these steps are catalyzed by NADP^+^‐dependent enzymes in case of FA chain elongation. The notion that the NAD^+^/NADH ratio in both mitochondria and peroxisomes is high with NAD^+^/NADH ratios of >10 and >500, respectively, explains the downhill oxidation of FAs in these two organelles whereas the high NADPH/NADP^+^ ratio (>1000 in the cytosol) is the driving force behind the uphill chain elongation of FA. The enzymology of the FACES has been resolved in recent years, with the first and third steps being catalyzed by multiple enzymes catalyzing the same chemical reaction but accepting different substrates,^23^ whereas the second and fourth steps are catalyzed by single enzymes. Indeed, the first step in the pathway, which is the condensation between an acyl‐CoA ester and malonyl‐CoA, is catalyzed by 7 different enzymes called elongases (ELOVL1–7), which are all integral membrane proteins localized in the ER. Although not resolved definitively, ELOVL2, 4, and 5 appear to be the ELOVLs playing a key role in PUFA biosynthesis (Figure 3), with ELOVL2 and ELOVL5 accepting different PUFA as substrates, whereas ELOVL4 accepts both saturated and unsaturated FA with a chain length of >24 carbon atoms regardless of the number of double bonds.^46^, ^47^ ELOVL4 is thus responsible for the production of both saturated as well as unsaturated VLC‐PUFA and ULC‐PUFA (Figure 3). ELOVL5 shows a preference for 18‐ and 20‐carbon PUFAs, whereas ELOVL2 is active with 20‐ and 22‐carbon PUFAs.^48^ ELOVL1, 3, and 7 are primarily, if not exclusively, involved in the formation of SFA and MUFA with chain lengths of up to 36 carbon atoms and more (Figure 3). The essential roles of the different elongases are stressed by the usually severe clinical signs and symptoms caused by the deletion of the various genes in mice (see Kihara^23^ for discussion) and variants in some of the *ELOVL* genes in humans, as discussed below. *Elovl1* KO mice, for instance, die within 1 day due to transepidermal water loss due to the marked reduction in VLC‐ceramides (>C26 carbon atoms), including ω‐*O*‐acylceramides.^49^ *Elovl4* KO mice lack most ω‐*O*‐acylceramides and >C28 non‐acylceramides and are neonatal lethal due to skin barrier dysfunction.^47^, ^50^ *Elovl2* KO mice are male infertile and have decreased ULC‐PUFAs,^51^ whereas *Elovl3* KO mice have a sparse haircoat and mild barrier defects.^52^

Remarkably, the second and fourth steps of the FA chain elongation cycle are catalyzed by single enzymes, which include the 3‐ketoacyl‐CoA reductase KAR and the *trans*‐2‐enoyl‐CoA reductase TECR (Figure 4). The third step in chain elongation, which involves the dehydration of 3‐hydroxyacyl‐CoAs, is catalyzed by one of four different enzymes, including HACD1/PTPLA, HACD2/PTPLB, HACD3/PTPLAD1, and HACD4/PTPLAD2.^53^ The specific role of each of these four enzymes has remained unclear which is especially true for HACD3 and HACD4. Nevertheless, the ubiquitously expressed enzyme HACD2 appears to be the major 3‐hydroxyacyl‐CoA dehydratase in humans. As discussed later, human HACD1 deficiency has been identified.

The result of all these FA modifications, followed by their activation to the corresponding acyl‐CoA esters in the case of 2‐OH‐FA and ω‐OH‐FA is an extensive pool of acyl‐CoA esters required for the synthesis of the large variety of lipids, which include glycerophospholipids, glycerolipids (mono‐, di‐, and triacylglycerols) and SL as the main classes of lipids. Below we describe the disorders of FA desaturation and chain elongation.

#### 2.4.1 FADS2 deficiency

The first patient affected by this deficiency was reported in 2001 as FA Δ^6^‐desaturase deficiency. The patient involved developed corneal ulcerations at a few weeks of life and developed hematemesis and feeding intolerance. Her subsequent clinical course was characterized by the persistence of corneal ulcerations, feeding intolerance, growth failure, and skin abnormalities.^44^ Since the FA profile in plasma from the patient showed some abnormalities, detailed studies in fibroblasts were initiated, which revealed that the conversion of linoleic acid (C18:2ω6) to AA (C20:4ω6) was grossly deficient in the patient's fibroblasts pointing to a deficiency in Δ^6^‐desaturation. Northern blot analysis demonstrated a profound decrease in Δ^6^‐desaturase mRNA. In 2003, the molecular defect in this patient was resolved by Nwankwo et al., who identified a nucleotide insertion in the transcriptional regulatory region of *FADS2* causing the decreased transcription of *FADS2*.^45^

#### 2.4.2 Acetyl‐CoA carboxylase A deficiency

Acetyl‐CoA carboxylases are biotin‐containing enzymes that catalyze the carboxylation of acetyl‐CoA to generate malonyl‐CoA. Humans express two different acetyl‐CoA carboxylases encoded by *ACACA* and *ACACB* which exert different roles in human metabolism. Indeed, the *ACACA* gene codes for the cytosolic enzyme ACC1 which provides the malonyl‐CoA required for FA chain elongation either by the FASN complex or the chain elongation system in the ER whereas the *ACACB* gene codes for the acetyl‐CoA carboxylase (ACC2) localized in the mitochondrial outer membrane which generates the malonyl‐CoA to control mitochondrial FA β‐oxidation (mFAO) by inhibiting the enzyme carnitine palmitoyltransferase (CPT1) which is also localized in the mitochondrial outer membrane.^54^ ACACA deficiency was first described by Lou et al. in a patient with global developmental delay, microcephaly, and dysmorphic facial features,^55^ followed by the identification of additional patients.^56^ ACACB deficiency has not been described so far.

#### 2.4.3 ELOVL1 deficiency

Dominant variants in ELOVL1 have been found to cause a similar phenotype characterized by pronounced ichthyotic keratoderma, spasticity, dysmorphic features, and early‐onset neurological disease with mild hypomyelination.^57^ The patients were diagnosed using whole exome sequencing, which revealed that both patients carried the same heterozygous variants in *ELOVL1*. The pathophysiological consequence of the p.Ser165Phe variant was tested in HEK293 cells, which revealed markedly reduced levels of C24:0–C28:0 as well as C26:1, with C26:0 being the most abnormal. These findings were fully in line with earlier work by Ohno et al.^58^ pointing to the key role of ELOVL1 in the provision of VLC‐acyl‐CoAs for SL synthesis. The same two patients were also studied by Mueller et al., who measured the enzymatic activity of the p.Ser165Phe variant, which turned out to be fully deficient.^59^ In addition, lipid analysis was performed, which revealed reduced C26‐ceramides and sphingomyelin, whereas C20‐ and C22‐sphingomyelins were increased. Altogether, these findings provide unequivocal evidence in favor of true ELOVL1 deficiency in these patients. Recently, Takahashi et al. described autosomal recessive variants in *ELOVL1* in two siblings with hypomyelinating spastic dyskinesia and ichthyosis caused by a homozygous splice site variant leading to exon skipping in *ELOVL1*.^60^

#### 2.4.4 ELOVL4 deficiency

This deficiency in FA chain elongation is associated with different clinical phenotypes and is inherited in both an autosomal recessive as well as autosomal dominant form. Indeed, in juvenile‐onset, autosomal dominant Stargardt macular dystrophy Type 3 (STGD3), a series of variants have been described in the last exon of *ELOVL4*, which appear to be causative for the loss of central vision with progressive degeneration of the macula and peripheral retina in patients. Like the autosomal recessive form of Stargardt disease (STGD1), the onset of loss of vision in patients ranges from 3 to 50 years, with a mean age of 14 years. Over decades, the macular lesion enlarges, and visual acuity decreases. The typical phenotype usually observed in patients is that of a well‐circumscribed homogeneous atrophy of the retinal pigment epithelium and choriocapillaris in the macula surrounded by yellow flecks and temporal optic nerve pallor. All the variants identified thus far in STGD3 patients cause a frameshift, giving rise to a premature stop codon and premature termination of the protein with detrimental consequences for the ELOVL4 enzyme because the shortened protein lacks the C‐terminal ER retention signal. Current evidence holds that the key pathogenetic mechanism is not haploinsufficiency of ELOVL4 as believed originally, but the dominant negative effect exerted by the mutant ELOVL4 protein, which traps wild‐type ELOVL4 as produced by the non‐mutated *ELOVL4* allele into perinuclear cytoplasmic inclusions which resemble “aggresomes.”^61^ Interestingly, biallelic variants in *ELOVL4* have been identified in patients with a completely different phenotype characterized by ichthyosis, seizures, mental retardation, and spasticity, which features resemble those observed in Sjögren–Larsson syndrome (SLS), although the neurological phenotype is more severe.^62^ The clinical phenotype associated with ELOVL4 deficiency has been expanded further by Cadieux‐Dion et al. who described a French–Canadian family with autosomal dominant spinocerebellar ataxia (SCA) and erythrokeratoderma^63^ and another report by Mir et al. who identified biallelic variants in three patients with neuro‐ichthyotic features from a consanguineous Pakistani family^64^ (for review, see Deák et al.^65^).

#### 2.4.5 ELOVL5 deficiency

This defect in FA chain elongation was first described in an Italian family with a pure form of SCA, which is a genetically heterogeneous group of autosomal dominant neurodegenerative disorders involving the cerebellum.^66^ Following the identification of a single missense variant in *ELOVL5* in this family, 456 independent SCA‐affected individuals were screened, which resulted in the identification of two unrelated families carrying the same p.Gly230Val variant. A more extensive analysis of the three families was published later.^67^ Since ELOVL5 is involved in the synthesis of PUFA of the ω3 and ω6 series, which includes AA and DHA, the levels of these two FA were measured in blood and found to be reduced to 30%–40% of normal.^66^ These findings prompted a double‐blind, randomized, placebo‐controlled trial in 10 ELOVL5 deficient patients who were supplemented with 600 mg of DHA per day for 16 weeks, followed by an open‐label study with overall 40 weeks of treatment. After 16 weeks of treatment, a mild but significant improvement of cerebellar functions was noted, which persisted over the full 40 weeks^66^ and 104 weeks^68^ period. Remarkably, no differences in serum DHA were found upon supplementation with DHA.

#### 2.4.6 3‐Hydroxyacyl‐CoA dehydratase deficiency

Humans express four different 3‐hydroxyacyl‐CoA dehydratases (*HACD1‐4*). HACD1 deficiency was first described in a single family with six affected individuals, all showing a severe myopathic phenotype at birth that gradually improved over time.^69^ In all patients, there was severe hypotonia in the neonatal period with head lag and absent tendon reflexes. Severe facial weakness was present in all patients, with drooling and reduced sucking reflexes in five of six patients. A marked delay in reaching milestones was apparent in all patients, which did improve with age, however, in all patients. Remarkably, cognition was within normal limits. Homozygosity mapping followed by exome sequencing revealed a nonsense variant in *HACD1*, causing premature termination of the HACD1 protein (p.Tyr258ter) and a full loss of enzyme activity. In recent years, additional patients have been identified.^70^, ^71^ No lipid analyses have been performed in any of these patients.

#### 2.4.7 *Trans*‐2,3‐enoyl‐CoA reductase deficiency

Thus far, only a single report has appeared that describes *trans*‐2,3‐enoyl‐CoA reductase (TER) deficiency in a consanguineous family with five young adults with non‐syndromic mental retardation.^72^ Three patients had an intention tremor and slow, rapid finger movements. There were no resting tremors, signs of ataxia, or other abnormal movements. Exome sequencing revealed a single variant in the *TERC* gene producing a p.Leu82Pro amino acid substitution in the TER enzyme. Subsequent work revealed that the mutant p.Leu82Pro protein is not only less stable but also catalytically less efficient. Furthermore, an abnormal SL profile was found in the patient's cells characterized by reduced C24 ceramide and sphingomyelin levels.^73^

## 3 FA HOMEOSTASIS AND THE RELEASE OF FA FROM VARIOUS LIPIDS

FA homeostasis dictates that all the FA incorporated into the various lipid species will be released again as part of the life cycle of each lipid species, after which the FA may join the cellular FA pool and are either reutilized in lipid synthesis or undergo oxidation via the different oxidation pathways in human cells including FA α‐oxidation in peroxisomes and FA β‐oxidation in peroxisomes and/or mitochondria as depicted in Figure 1. The mechanisms involved in the release of FA from the various lipids are different for each of the different lipid classes.

### 3.1 Triacylglycerols

Triacylglycerols or triglycerides are synthesized at the ER membrane via the stepwise addition of three acyl‐CoAs mediated by the enzymes glycerol‐3‐phosphate acyltransferase, 1‐acylglycerol‐3‐phosphate *O*‐acyltransferase, phosphatidate phosphatase, and diacylglycerol *O*‐acyltransferase and then packaged into lipid droplets surrounded by a monolayer membrane and released into the cytosol. Lipolysis is low under certain well‐fed conditions but is induced by β‐adrenergic stimulation upon fasting and requires the activity of three lipases, notably adipose triglyceride lipase, hormone‐sensitive lipase, and monoacylglycerol lipase, at least in the adipocyte.^74^ The FA released may have several destinations, including their oxidation in peroxisomes and/or mitochondria. In recent years it has become clear that contact zones between lipid droplets and the various subcellular compartments within a cell play a crucial role in the trafficking of FA and their subsequent metabolism. Indeed, lipid droplets do not only interact with lysosomes (lipophagy) and the ER (lipid droplet biosynthesis and extension), but also with peroxisomes and mitochondria. This contact between organelles is mediated by so‐called tethering proteins. Work by Chang et al.^75^ has shown that the hereditary paraplegia protein M1 Spastin, a membrane‐bound AAA‐ATPase found on lipid droplets, coordinates FA trafficking from lipid droplets to peroxisomes by forming a tethering complex with the peroxisomal ABCD1 protein which is defective in X‐linked adrenoleukodystrophy (ALD) patients, as will be discussed later. This M1 Spastin‐ABCD1 partnership ensures the effective channeling of FA from lipid droplets to peroxisomes for FA oxidation. Whereas the bulk of triacylglycerols possess ester bonds at the *sn‐1*, *sn‐2*, and *sn‐3*‐position, in some triacylglycerols the side chain is attached to the glycerol backbone via an ether linkage (see Section 3.5).

### 3.2 Sphingolipids

The degradation of all SL, including gangliosides, sulfatides, and sphingomyelin, proceeds via ceramide as a central metabolite. Indeed, SL are all converted back into ceramide through the stepwise removal of the various headgroup components attached to the ceramide backbone which occurs notably in lysosomes through the concerted action of the various lysosomal hydrolases (Figure 2 and Pant et al.^76^). Subsequently, the *N*‐acyl group in ceramide is removed by one of five different ceramidases which include the acid ceramidase ASAH1/AC, the neutral ceramidase ASAH2/NC, and the alkaline ceramidases 1–3 (ACER1, ACER2, ACER3) of which the lysosomal ceramidase ASAH1 is probably the most important as exemplified by the clinical consequences of ASAH1 deficiency (Farber disease). The LCB produced in the ceramidase reaction are then phosphorylated by the two redundant sphingosine kinases SPHK1 and SPHK2, which accept not only the major LCB sphingosine as substrate but also the minor LCB, that is, dihydrosphingosine and phytosphingosine, as substrate.^23^ The different phosphorylated LCB are then cleaved by the enzyme sphingosine‐1‐phosphate (S1P) lyase, an integral ER membrane protein^23^, ^77^ into the different aldehydes (*trans*‐2‐hexadecenal from S1P, hexadecanal from dihydro‐S1P, and 2‐OH‐hexadecanal from phyto‐S1P) plus phosphoethanolamine.^23^ The fatty aldehydes produced in the S1P‐lyase reaction are then converted into the different FA, including C16:0, C16:1, and 2‐OH‐C16:0, which is mediated by members of the ALDH family (19 in humans) notably by the fatty aldehyde dehydrogenase FALDH, encoded by *ALDH3A2* which is the enzyme deficient in SLS. The FA produced by FALDH then joins the cellular FFA pool (Figures 1 and 2A).

### 3.3 Glycerophospholipids

Glycerophospholipids are predominantly incorporated in the various cellular and subcellular membranes, which all have a different composition, with some phospholipids confined to only a single membrane, like cardiolipins in mitochondrial membranes. The FA contained in glycerophospholipids are released from the glycerol backbone by different members of the phospholipase family and the FA released can either be used for the synthesis of other metabolites, including the many bioactive lipids generated from AA, EPA, and DHA (see Figure 3 and Dyall et al.^5^) or enter the FFA pool to undergo oxidation or be reutilized again (Figure 1).

### 3.4 Cholesterol esters

Cholesterol esters are synthesized from free cholesterol and different acyl‐CoAs by one of several cholesterol acyltransferases and subsequently hydrolyzed again by different esterases, including the lysosomal cholesterol esterase deficient in Wolman disease.

### 3.5 Ether(phospho)lipids

Ether‐linked glycero(phospho)lipids differ from the regular glycero(phospho)lipids in one major aspect, which is the fact that the side chain is attached to the *sn‐1* position of the glycerol backbone via an ether linkage instead of an ester linkage. In humans, the ether linkage in most ether lipids contains a double bond immediately adjacent to the ether linkage (an alk‐1‐enyl ether) and thus have the 1‐alkenyl‐2‐acyl configuration instead of the 1‐alkyl‐2‐acyl configuration in regular ether(phospho)lipids. The headgroup in ether phospholipids is either phosphoethanolamine or phosphocholine. 1‐Alkenyl‐2‐acylphospholipids are better known as “plasmalogens” and are predominantly components of membrane bilayers, accounting for about 18% of total phospholipids in humans. They are especially abundant in the brain (myelin) and also other organs, including the heart and the skeletal muscle, but not the liver.^78^ Importantly, the fatty alcohol required for the synthesis of the 1‐alk(en)yl bond is synthesized by two acyl‐CoA:NADPH oxidoreductases FAR1 and FAR2.^79^ The finding that ether lipid synthesis is deficient in patients with FAR1 deficiency stresses the importance of FAR1 in humans.^80^ The recent identification of patients with paraparesis and bilateral cataracts caused by heterozygous de novo variants in *FAR1* resulting in uncontrolled synthesis of ether lipids, stresses the importance of homeostasis, which in this case involves the tight control of ether lipid biosynthesis.^81^ The breakdown of ether(phospho)lipids is less well understood. Recent work by van Wouw et al., however, has shed new light on this aspect of ether(phospho)lipid homeostasis.^82^ Indeed, they discovered that the sterol‐regulated transmembrane protein TMEM86a, which is a direct target of the nuclear hormone LXR, displays lysoplasmalogenase activity, thus releasing the 1‐alkenyl group of lysoplasmalogens as fatty aldehyde which will then be converted into the corresponding FA most likely via FALDH (Figure 1). The many disorders associated with the release of FA from all these different lipid species will not be discussed here.

## 4 FA HOMEOSTASIS AND THE DEGRADATION OF FA BY THE PEROXISOMAL AND MITOCHONDRIAL FA OXIDATION SYSTEMS

FA homeostasis requires that the FA derived from exogenous, dietary sources or synthesized de novo, followed by their modification into other FA, are also degraded again to keep the intracellular FA pool in check. A minor alternative pathway through which FFA may be withdrawn from the intracellular FA pool without undergoing oxidation involves their conversion into some other FA species, which are then excreted out of the cell into the plasma compartment and leave the human body via the urine and/or stools. A good example of the latter FA homeostatic pathway concerns the conversion of acyl‐CoAs into the corresponding acylcarnitines via one of the intracellular carnitine acyltransferases. Indeed, acyl‐CoAs cannot leave the cell themselves but can do so after conversion into acylcarnitines, which can then leave the cell and, in fact, the human body. Other examples of this non‐oxidative FA homeostatic mechanism include the conversion of acyl‐CoAs into glycine and taurine esters. Although these non‐oxidative mechanisms are physiologically relevant, it is the oxidation of FA via the peroxisomal and mitochondrial FA oxidation systems which contribute most to FA homeostasis. We will briefly discuss the different FA oxidation systems below.

### 4.1 Peroxisomal FA β‐oxidation

**FIGURE 5:**
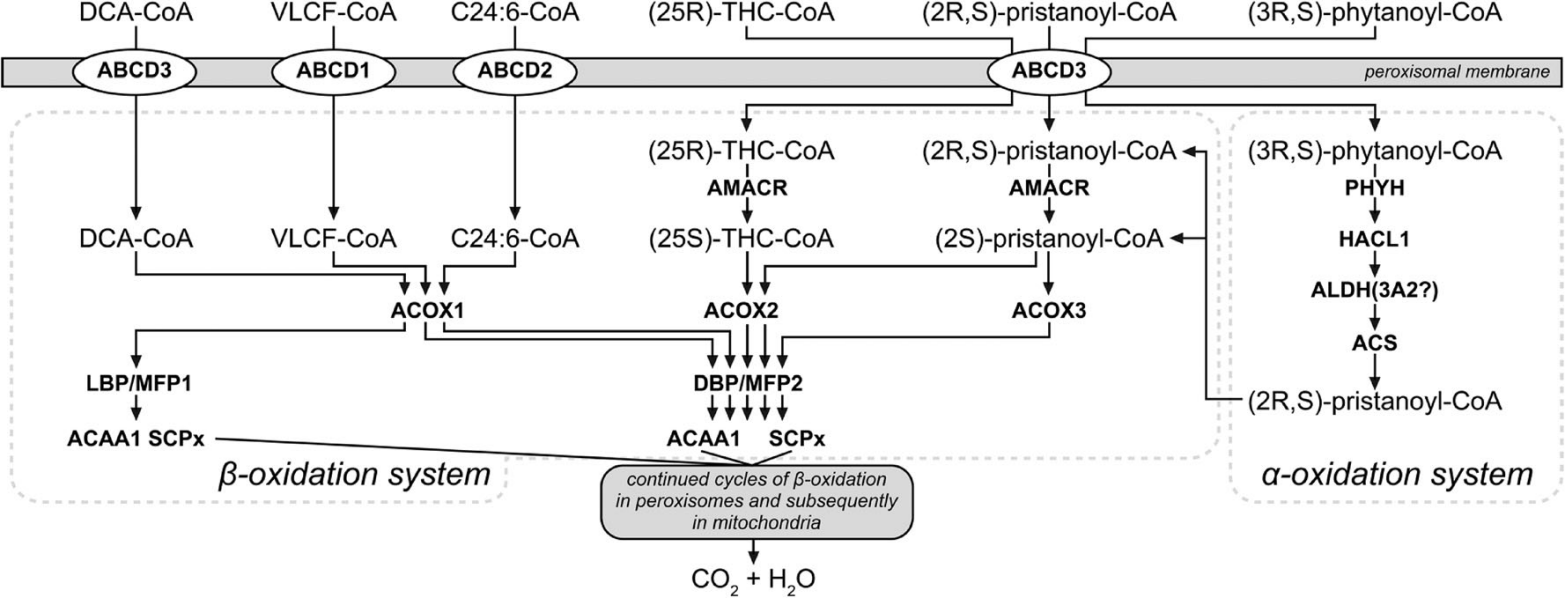
Schematic representation of the peroxisomal FA α‐ and β‐oxidation systems. Fatty acids such as very long‐chain fatty acids (VLCF‐CoA), di‐ and trihydroxycholestanoic acids (DHCA/THCA), pristanic acid, dicarboxylic acids, as well as tetracosahexaenoic and tetracosapentaenoic acids (C24:6ω3 and C24:5ω3), are imported via ABCD transporters and subsequently shortened in a series of reactions. The initial dehydrogenation step is catalyzed by ACOX1, 2, and 3, each with a different substrate specificity. Further processing involves the bifunctional enzymes LBP/DBP and the thiolases ACAA1 and SCPx. Distinct enzymes of the β‐oxidation system are utilized for different substrates, with 2‐methyl‐acyl‐CoA racemase (AMACR) playing a crucial role in the racemization of pristanic acid as well as DHCA and THCA, the intermediates in bile acid synthesis, preparing them for subsequent β‐oxidation steps. The α‐oxidation pathway is reserved for the conversion of (activated) phytanic acid into pristanic acid allowing the latter to enter the β‐oxidation system.

Peroxisomes are not so important for the degradation of LCFA and MCFA, which are predominantly oxidized in mitochondria, but they do play a crucial role in cellular FA oxidation because several FA can only be degraded in peroxisomes and not in mitochondria. From the perspective of human diseases, the most important FA oxidized in peroxisomes are (a) VLCFA, notably C26:0; (b) di‐ and trihydroxycholestanoic acid (DHCA and THCA), which are key intermediates in the biosynthesis of the respective primary bile acids chenodeoxycholic acid and cholic acid from cholesterol; (c) pristanic acid derived from dietary sources either directly or indirectly from phytanic acid; (d) tetracosahexaenoic acid and tetracosapentaenoic acid (C24:6ω3 and C24:5ω3), which are key intermediates in the biosynthesis of DHA and docosapentaenoic acid (C22:6ω3 and C22:5ω3); and (e) long‐chain dicarboxylic acids (Figure 5; for recent review Wanders et al.^83^).

The basic machinery involved with the oxidation of FA in peroxisomes is identical to that in mitochondria, although there are important differences between the two systems (Figure 5). The first step in peroxisomal β‐oxidation is catalyzed by one of three different acyl‐CoA oxidases (ACOX1, 2, and 3), which use molecular oxygen as an electron donor rather than electron transfer flavoprotein (ETF) as used by the mFAO enzymes. The second and third steps in peroxisomal β‐oxidation are catalyzed by two so‐called bifunctional proteins (LBP and DBP), which exhibit different substrate specificities, whereas the last step of peroxisomal β‐oxidation is catalyzed by one of two different thiolases (ACAA1 and SCPx) each reactive with a different set of substrates.^84^ Importantly, the substrates of the peroxisomal β‐oxidation machinery are all handled by D‐bifunctional protein (DBP) with one notable exception, the long‐chain dicarboxylic acids.^85^ Needless to say, the peroxisomal and mitochondrial β‐oxidation enzymes are all encoded by different genes (see Wanders et al.^86^ for details). Another important difference between the mitochondrial and peroxisomal β‐oxidation systems concerns the import of FA from the cytosol. For mitochondria, this is mediated by the mitochondrial carnitine cycle, whereas peroxisomes acquire their FA substrates from the cytosol not as acylcarnitine esters but as acyl‐CoAs, which are transported across the peroxisomal membrane via one of the three ABCD transporters (ABCD1, 2, and 3).^87^ It is also important to realize that the peroxisomal β‐oxidation system is only a chain‐shortening system simply because the different acyl‐CoA oxidases do not accept short‐chain acyl‐CoAs as substrate. This implies that the products of peroxisomal β‐oxidation are acetyl‐CoA, propionyl‐CoA, and different medium‐chain acyl‐CoAs, which are then transferred to mitochondria to undergo full oxidation to CO_2_ and H_2_O via one of two different mechanisms.^88^

The peroxisomal FA β‐oxidation deficiencies: Among the disorders of peroxisomal FA, β‐oxidation X‐linked ALD is most frequent, with a birth prevalence of 1:15 000 male plus female patients.^89^ Below, we will describe these disorders in decreasing order of frequency.

#### 4.1.1 X‐linked ALD

ALD is a relatively rare neurometabolic disorder caused by pathogenic variants in *ABCD1* and is characterized by a challenging phenotypic diversity including (1) a slowly progressive myeloneuropathy; (2) a rapidly progressive inflammatory leukodystrophy (cerebral ALD) and (3) primary adrenal insufficiency. These three core clinical syndromes are not present in all patients and appear unrelated to genotype. Cerebral ALD and adrenal insufficiency require early detection, followed by clinical surveillance and prompt intervention when needed. Hematopoietic cell transplantation is the treatment of choice for cerebral ALD patients despite the fact that autologous hematopoietic stem cell transplantation after ex vivo lentiviral gene therapy has been described as a safe alternative.^90^ However, this treatment option is not generally available at present. Furthermore, no safety data are available yet. The need for early detection of ALD patients has prompted the inclusion of ALD in neonatal screening programs, at least in several states in the USA, using C26:0‐lysophosphatidylcholine (C26:0‐lysoPC) as a biomarker. In the Netherlands ALD has just been included in the Dutch neonatal screening program (start October 2, 2023) using a somewhat different sex‐specific protocol.^91^ Recently, a consensus‐based modified Delphi approach involving 28 international ALD experts was used to develop best‐practice recommendations for diagnosis, clinical surveillance, and treatment of patients with ALD.^92^ The fact that transplantation‐based procedures are suitable for only a small group of patients because of the mortality and morbidity associated with myeloablative procedures and the problem of identifying suitable donors, particularly in adults, has inspired the search for alternative options. In this respect, the recent work by Köhler et al. must be mentioned, which involved a randomized, double‐blind, multicenter, placebo‐controlled phase 2–3 trial in which the safety and efficacy of leriglitazone was tested.^93^ Leriglitazone is a novel, neuroprotective brain‐penetrant PPARγ agonist which affects the expression of multiple genes, ultimately resulting in decreased oxidative stress, reduced inflammation, and protection of the blood–brain barrier against disruption. The finding that cerebral ALD, which is a life‐threatening event in adrenomyeloneuropathy patients, occurred only in the placebo group raises hope that leriglitazone may at least slow the progression of cerebral ALD.

#### 4.1.2 DBP deficiency

The clinical signs and symptoms of DBP deficiency caused by variants in *HSD17B4* are, in general, very severe and resemble those observed in patients affected by a Zellweger spectrum disorder (ZSD), which are caused by pathogenic variants in genes coding for proteins involved in peroxisome biogenesis.^94^, ^95^ This even includes the MRI features of DBP‐deficient patients.^96^ We have earlier reported the clinical, biochemical, and genetic findings in a cohort of 126 patients.^97^ This survey revealed that the clinical presentation is dominated by neonatal hypotonia (98%) combined with seizures within the first few months of life (93%) and failure to thrive (43%). Most patients had dysmorphic features, including macrocephaly, high forehead, flat nasal bridge, low set ears, large anterior fontanelle, and micrognathia. Only a few patients acquired any significant milestones, and the few that did show subsequent loss of motor achievements. DBP deficiency can be classified into three different subgroups, but the clinical phenotype associated with any of these three are indistinguishable, and the same applies to their prognosis.^97^ In more recent years, several patients have been identified mostly through whole exome/genome sequencing with much milder phenotypes, including Perrault syndrome.^98^, ^99^ Since DBP is involved in the β‐oxidation of basically all peroxisomal FA substrates except long‐chain dicarboxylic acids,^100^ typical patients show the accumulation of VLCFA, the bile acid intermediates DHCA and THCA, as well as pristanic acid and (secondary) phytanic acid. However, in literature, several patients have been described with an incomplete peroxisomal biomarker profile, which even includes normal VLCFA, thus warranting caution in the interpretation of laboratory metabolite analyses.^101^ The recent discovery that C26:0‐lysoPC is a superior biomarker compared to VLCFA analysis—at least for ALD, as discussed above—may resolve this potential diagnostic pitfall in the future.

#### 4.1.3 ACOX1 deficiency

ACOX1 deficiency was first described in 1988 in two patients originally supposed to suffer from a milder form of Zellweger syndrome, but in whom peroxisomes were found to be normally present‐albeit enlarged‐in the patients' liver biopsies, ACOX1 deficiency has so far been described in some 30–40 patients. In 2007 we reported the clinical, biochemical, and genetic characteristics of a group of 22 ACOX1‐deficient patients,^102^ which revealed that most patients exhibited neonatal‐onset hypotonia, seizures, failure to thrive, psychomotor retardation, sensorineural hearing loss, hepatomegaly, and visual loss with retinopathy. In 50% of the patients, dysmorphic features were present, resembling those observed in ZSD patients. In some patients, there was some early motor development, but they typically regressed by 2–3 years. In more recent years—again thanks to whole exome/genome sequencing—more milder forms of ACOX1 deficiency have been described. This includes two adult patients (brothers) with normal early development who developed progressive neurological symptoms only in later childhood.^102^ In ACOX1‐deficient patients there is only VLCFA accumulation with normal levels of the other peroxisomal biomarkers. In some patients, VLCFA levels are only minimally abnormal, which complicates the laboratory diagnosis of ACOX1 deficiency. Future will tell whether the analysis of C26:0‐lysoPC may prove to be a better biomarker as established for ALD.

#### 4.1.4 ACOX2 deficiency

ACOX2 deficiency was identified independently by two groups of investigators in two patients showing markedly different clinical features. Indeed, the patient identified by Monte et al. involved a 16‐year‐old male with elevated transaminases (twofold to fivefold upper normal limit) and no other symptoms.^103^ In contrast, the patient described by Vilarinho et al. had a much more severe phenotype characterized by liver fibrosis, mild ataxia, and cognitive impairment with intermittently elevated transaminases and died in early childhood.^104^ The patient identified by Ferdinandusse et al. also showed severe clinical features.^105^ In all three patients, there was an accumulation of the C_27_‐bile acid intermediates DHCA and THCA in line with the crucial role of ACOX2 in bile acid synthesis (see Figure 5). All the other peroxisomal biomarkers in plasma were normal. Interestingly, Alonso‐Peña et al. recently studied a group of 33 patients with unexplained hypertransaminasemia, which led to the identification of ACOX2 deficiency in 4 of the 33 patients, as concluded from molecular and biochemical analyses. All patients had minimal clinical signs and symptoms. Nevertheless, all patients were treated with ursodeoxycholic acid, which resulted in lower C_27_‐bile acid levels and relief of symptoms.^106^

#### 4.1.5 2‐Methyl‐acyl‐CoA racemase deficiency

Since the first description of 2‐methyl‐acyl‐CoA racemase (AMACR) deficiency in patients affected by an adult‐onset motor neuropathy,^107^ 15 additional patients with this defect have been described in the literature as recently summarized by Tanti et al.^108^ The enzyme AMACR is one of the auxiliary enzymes required for the β‐oxidation of a subset of FA oxidized in peroxisomes (see Figure 5) which includes pristanic acid, DHCA and THCA but not VLCFA. The signs and symptoms of these patients were markedly different, ranging from a severe phenotype characterized by fulminant liver failure very early in life,^109^ which required liver transplantation, to much milder phenotypes.^110^, ^111^ Recently, Tanti et al. reported a female patient presenting at an older age with episodes of dysphasia, headache, and sensory disturbances with unusual changes observed upon MRI, which prompted suspicion of mitochondrial disease, but extensive testing did not identify the cause of her disorder. After 8 years of symptoms, the patient developed a febrile encephalopathy with hemispheric dysfunction, focal convulsive seizures, and coma. The diagnosis remained elusive until whole exome sequencing pointed to AMACR deficiency.

#### 4.1.6 Contiguous ABCD1, DXS1357A deletion syndrome

Contiguous ABCD1, DXS1357A deletion syndrome was first described in 2002 in three newborns with clinical signs and symptoms suggestive of a ZSD.^112^ The patients—all male—suffered from motor and intellectual disabilities, dystonia, sensory neural deafness, and white matter changes. Plasma VLCFA was increased in all three patients. Detailed studies in the patients' fibroblasts revealed that peroxisome biogenesis was normal and that the defect in these patients was confined to the oxidation of VLCFA since all other peroxisomal markers were normal. These puzzling findings were resolved by the identification of a small deletion in the Xq28 region spanning the 5′ ends of *ABCD1* and *DXS1357E*/*BCAP31*, which codes for BAP31, an abundant ER protein of unresolved function. *ABCD1* and *BCAP31* are located head‐to‐head at Xq28. Several additional patients have been described in the literature which includes a patient with a larger deletion extending to *SLC6A8* encoding the creatine transporter.^113^, ^114^

#### 4.1.7 Sterol‐carrier‐protein X deficiency

After its first description in 2006 in a patient with leukoencephalopathy with dystonia and motor neuropathy,^115^ only a few patients have since been described. The second patient was reported by Horvath et al. and involved a 51‐year‐old male presenting with adult‐onset SCA and brain MRI abnormalities characteristic of NBIA.^116^ Pristanic acid was markedly elevated in the patient's plasma, whereas phytanic acid was only mildly elevated. Plasma VLCFA were normal. This prompted molecular analysis, which revealed two variants of unknown significance in the SCP2 gene. Subsequent immunoblot analyses revealed the virtual absence of the 58 kDa SCPx protein, confirming SCPx deficiency. An unusual retinal phenotype was later described in the same patient.^117^ A third patient with presumed SCPx deficiency was recently reported by Galano et al., but it remains to be established whether this patient is truly affected by SCPx deficiency.^118^

#### 4.1.8 ABCD3 (PMP70) deficiency

This defect has thus far only been described in a single patient who presented with hepatosplenomegaly and severe liver disease, which progressed to the extent that liver transplantation was required at 4 years of age. The levels of THCA and DHCA were elevated in the patient's plasma, which is in line with the role of ABCD3 in the peroxisomal β‐oxidation system. The patient expired shortly after transplantation.^119^

#### 4.1.9 Acyl‐CoA binding protein 5 deficiency

Acyl‐CoA binding protein 5 (ACBD5) deficiency was first described in 2013 when Abu‐Safieh et al. performed molecular analyses in a large cohort of 150 simplex as well as multiplex patients affected by different retinal dystrophy (RD) phenotypes. Apart from many known RD genes, this study identified six new RD genes of which two were associated with novel syndromic forms of RD.^120^ One of these newly identified genes was *ACBD5*. The realization that ACBD5 deficiency belongs to the group of peroxisomal disorders only came later in 2017 thanks to the work of Yagita et al.,^121^ who performed studies in the original patients identified by Abu‐Safieh et al. and our own work.^122^ The patient we described was suspected to suffer from a peroxisomal disorder based on clinical signs and symptoms, which included progressive leukodystrophy, syndromic cleft palate, ataxia, and RD. Subsequent analysis of peroxisomal metabolites in plasma revealed elevated VLCFA levels with no abnormalities in any of the other peroxisomal biomarkers in plasma. Follow‐up studies in the patient's fibroblasts showed reduced VLFCA β‐oxidation, which was not due to a deficiency of one of the known β‐oxidation enzymes but instead of ACBD5, which is involved in the transfer of VLFCA from its site of synthesis, that is, the ER to the peroxisome.^122^ Since these early reports, several additional patients with ACBD5 deficiency have been described with novel clinical features that extend the phenotypic spectrum associated with ACBD5 deficiency.^123^, ^124^, ^125^

### 4.2 Peroxisomal α‐oxidation

Certain FAs cannot be degraded by β‐oxidation right away but require auxiliary steps to modify the FA in such a way that they can undergo β‐oxidation. This includes 3‐methyl‐branched‐chain FA, which first requires oxidative decarboxylation to turn the 3‐methyl FA into the corresponding 2‐methyl FA. The latter FA can be oxidized by β‐oxidation. Phytanic acid (3,7,11,15‐tetramethylhexadecanoic acid) is the most important 3‐methyl FA, which is converted via oxidative decarboxylation, also called α‐oxidation, into the 2‐methyl FA pristanic acid (2,6,10,14‐tetramethylpentadecanoic acid). The oxidative decarboxylation of 3‐methyl FAs is not mediated by a single enzyme but involves a complex 5‐enzyme pathway (see Figure 5) with PHYH as the key enzyme.^13^ Refsum disease is the only disorder of peroxisomal α‐oxidation identified so far and is caused by biallelic variants in the gene encoding *PHYH* as discussed below.

Other examples of FA that require auxiliary steps before they can undergo β‐oxidation are unsaturated acyl‐CoAs whose oxidation depends on dienoyl‐CoA reductases and enoyl‐CoA isomerases to reposition the double bond, but also FA with a 2‐methyl group at the (2R)‐position, like pristanic acid, that require the active participation of the enzyme AMACR.

#### 4.2.1 The peroxisomal FA α‐oxidation disorders

Adult Refsum disease (ARD), as caused by biallelic variants in the gene coding for PHYH, is so far the only disorder of peroxisomal α‐oxidation.^126^, ^127^ Affected patients are asymptomatic at birth, show no obvious defects in growth and development, and are usually present in late childhood with progressive loss of night vision, a decline in visual acuity, and anosmia. Over the years, patients usually develop additional abnormalities, including deafness, demyelinating polyneuropathy, ichthyosis, fatigue, and cardiac conduction disturbances.^128^ The full constellation of signs and symptoms originally defined by Refsum in the 1940s, which includes retinitis pigmentosa, cerebellar ataxia, and chronic polyneuropathy, is rarely seen in single patients. According to Wierzbicki et al., who studied a cohort of 15 ARD patients over many years, loss of vision (15/15) and anosmia (14/15) are the two dominant features observed in patients.^128^ The Polyneuropathy is of a mixed motor and sensory type, which is asymmetrical, chronic, and progressive in untreated ARD patients. Over the years, muscular weakness can become widespread, affecting the limbs but also the trunk. Some patients develop cardiomyopathy, which may be lethal. The only peroxisomal biomarker that is abnormal in ARD is phytanic acid, which is usually markedly elevated in patients reaching levels beyond 1000 μmol/L (normal: <10 μmol/L). Plasma phytanic acid is a very robust biomarker for ARD with no false negatives reported so far.

ARD is one of the few peroxisomal disorders amenable to therapy. Indeed, dietary restriction of phytanic acid is extremely important and rewarding since several features may stabilize or even improve, including peripheral neuropathy, ataxia, ichthyosis, and cardiac features. However, the retinitis pigmentosa, deafness, and anosmia appear to be more refractory. ARD can also be caused by partial loss of function variants in *PEX7*, which also results in defective peroxisomal α‐oxidation because of the deficient import of PHYH into the peroxisome.^129^

### 4.3 Mitochondrial FA β‐oxidation

The mFAO system plays a central role in intracellular FA homeostasis, as exemplified by the multiple FA disturbances in patients affected by a defect in one or more of the enzymes and transporters involved. The mitochondrial FA oxidation system differs from the peroxisomal system in many respects, although the basic architecture of the two systems is identical and involves four subsequent steps to remove an acetyl‐CoA unit from the various acyl‐CoAs. Each of these four enzymatic reactions is catalyzed by multiple enzymes, each having its own substrate specificity. The first step in mitochondrial FA oxidation, for instance, is catalyzed by at least three different acyl‐CoA dehydrogenases specifically reacting with long‐chain, medium‐chain, and short‐chain acyl‐CoAs (i.e., VLCAD, MCAD, and SCAD) and the same is true for the other three steps of β‐oxidation. Another major difference between the two systems is that the reducing equivalents contained in FADH_2_ and NADH as produced upon mitochondrial β‐oxidation are directly fed into the respiratory chain to generate ATP in contrast to the situation in peroxisomes, which lack a respiratory chain. Furthermore, the acetyl‐CoA generated by mitochondrial β‐oxidation is directly oxidized to CO_2_ and H_2_O in the mitochondrial citric acid cycle. Since peroxisomes lack a citric acid cycle, the acetyl‐CoA produced in peroxisomes cannot be degraded to CO_2_ and H_2_O within peroxisomes but is instead transferred to mitochondria either as acetylcarnitine or acetate followed by oxidation to CO_2_ and H_2_O in mitochondria.

#### 4.3.1 The mitochondrial FA oxidation disorders

mFAO can be deficient due to biallelic mutations in genes coding for one of the structural β‐oxidation enzymes catalyzing the actual FA β‐oxidation (the primary mFAO deficiencies) but can also be impaired because of other factors affecting FA β‐oxidation—either genetic in origin or not—for instance, because of mutations in genes coding for different enzymes or transporters not directly involved in mFAO per se. The prototype of these secondary mFAO disorders is glutaric aciduria Type II, better known as multiple acyl‐CoA dehydrogenase deficiency, as caused by defects in the ETF system. Other examples of secondary mFAO deficiencies are riboflavin transporter defects,^130^ FAD synthase deficiency,^131^ and mitochondrial FAD transporter deficiency.^132^ The primary mFAO deficiencies are usually subdivided into two groups, including the disorders of long‐chain and medium‐chain/short‐chain FA. The latter group consists of medium‐chain acyl‐CoA dehydrogenase (MCAD), short‐chain acyl‐CoA dehydrogenase (SCAD), and short‐chain 3‐hydroxyacyl‐CoA dehydrogenase (SCHAD) deficiency, whereas the first group includes carnitine palmitoyl transferase 1A (CPT1A), carnitine acylcarnitine translocase (CACT), carnitine palmitoyltransferase 2 (CPT2), very long‐chain acyl‐CoA dehydrogenase (VLCAD), and long‐chain 3‐hydroxyacyl‐CoA dehydrogenase/mitochondrial trifunctional protein (LCHAD/MTP) deficiency.

A common characteristic of all mFAO deficiencies is hypoketotic hypoglycemia caused by the enhanced reliance on glucose oxidation in case mFAO is blocked. The hypoketotic hypoglycemia can be life‐threatening, especially when glycogen reserves are low, giving rise to early death if not recognized in time. This is true for all mFAO disorders, including MCAD deficiency. The fact that prevention of hypoglycemia and timely treatment leads to improved prognosis is one of the main reasons why mFAO disorders have been included in neonatal screening programs around the world.

In line with the important role of mFAO in the heart and in skeletal muscle, mFAO‐deficient patients may suffer from cardiac and skeletal muscle abnormalities, especially in severe cases with low residual enzyme activities. Indeed, hypertrophic and dilated cardiomyopathy are frequent findings in patients with deficiencies at the level of CACT, CPT2, VLCAD, and LCHAD/MTP, but not CPT1A, which follows logically from the fact that CPT1B and not CPT1A is the predominant CPT expressed in heart muscle. Arrhythmias and conduction defects are also frequently observed in these patients. Indeed, in a study of 107 mFAO‐deficient patients, cardiac involvement was found in >50% of patients: 67% of these patients presented with cardiomyopathy (mostly hypertrophic), and 47% had heartbeat disorders with various conduction abnormalities and arrhythmias associated with collapse, near‐miss, and sudden unexpected deaths,^133^ (see also Baruteau et al.^134^). All enzymatic deficiencies except CPT1A and MCAD deficiency were found to be associated with cardiac signs. Muscular signs and symptoms were observed in 51% of patients of whom 64% had myalgias or paroxysmal myoglobinuria and 29% had progressive proximal myopathy. Chronic neurological presentations are rare except in patients with LCHAD/MTP deficiency in whom peripheral neuropathy and retinitis pigmentosa are frequent findings.^135^ Hepatomegaly and hepatic features are also observed in mFAO‐deficient patients, albeit less frequently (for more detailed information, see Wanders and colleagues^86^, ^136^, ^137^, ^138^). In MCAD deficiency, FA oxidation is only partially deficient since LCFA can be broken down to MCFA up to the level of C8–C10, which explains why MCAD deficiency is much less severe compared to long‐chain mFAO defects. Furthermore, MCFA are much less toxic per se compared with LCFA. Nevertheless, the hypoglycemia in MCAD deficiency can be life‐threatening and may lead to early death, which explains its inclusion in neonatal screening programs.

In all mFAO disorders except CPT1A deficiency, there is an intracellular accumulation of acyl‐CoA esters mostly within mitochondria, again with one exception, which is CACT deficiency. Acyl‐CoA esters are not able to pass membranes as such but can only transfer from one compartment to the other after prior conversion into the corresponding acylcarnitine via one of the intracellular carnitine acyltransferases. These acylcarnitines are not only able to traverse the mitochondrial membrane but also the plasma membrane to end up in the plasma compartment. This phenomenon has rendered plasma acylcarnitine analysis an excellent tool in the diagnosis of mFAO patients with characteristic acylcarnitine profiles for each of the mFAO enzyme deficiencies (see Wanders et al.^86^). Recent lipidomics studies have revealed that the lipidome of fibroblasts from mFAO‐deficient patients is changed including the highly increased levels of lysophospholipids and plasmalogens and a profound remodeling of cardiolipin species.^139^ Furthermore, tracer‐based lipidomics has enabled the discovery of disease‐specific lipid biomarkers different from acylcarnitines, which include a specific lysophosphatidylcholine (LPC (14:1)) in VLCAD deficiency and *S*‐(3‐hydroxyacyl)cysteamines for LCHAD deficiency.^140^

## 5 CONCLUDING REMARKS

In summary, much has been learned in recent years about the homeostasis of FA and, the defects in humans and the many genetic defects causing impairments in FA homeostasis due to pathogenic variants in genes coding for enzymes involved in FA homeostasis. In the last decade, patients were more frequently identified by genetic testing, whereas previously, biochemical approaches were predominant. The rapid developments in metabolomics and, for this field, especially, lipidomics will certainly contribute to the diagnostic process to provide biochemical read outs. These will not only contribute to the interpretation of variants found in patients, but also will open avenues to gain further insight into pathophysiology and potentially even treatment.

## FUNDING INFORMATION

The authors have no financial support to declare relevant to this study.

## CONFLICT OF INETEREST

The authors declare no competing interests.
